# Exploring determinants of flourishing: a comprehensive network analysis of retirees in Taiwan

**DOI:** 10.1186/s12889-024-19466-x

**Published:** 2024-07-19

**Authors:** Wan-Chen Hsu, Nuan-Ching Huang, Chung-Lin Li, Susan C. Hu

**Affiliations:** 1 Department of Public Health, College of Medicine, National Cheng Kung University, Tainan, Taiwan; 2 Healthy Cities Research Center, Innovation Headquarters, National Cheng Kung University, Tainan, Taiwan; 3 Department of Public Health, College of Medicine, National Cheng Kung University, Tainan, Taiwan

**Keywords:** Human flourishing, Network analysis, Retiree, Well-being

## Abstract

**Background:**

Human flourishing is an emerging concept, extending beyond the conventional boundaries of subjective well-being and evolving into a comprehensive capture of the diverse dimensions of human life within complex societal structures. Therefore, moving away from traditional approaches centered on the single latent construct, this study aims to explore the multiple aspects of human flourishing and the intricate interplay of their contributing factors.

**Methods:**

Data were collected from the Health and Living Environments Survey of Taiwanese Retirees during 2023 (valid sample *n* = 1,111). Human flourishing was measured using the Secure Flourish Index developed by Harvard University, which includes 12 indicators: (1) life satisfaction, (2) happiness, (3) mental health, (4) physical health, (5) meaning in life, (6) sense of purpose, (7) promoting good, (8) delaying gratification, (9) content relationships, (10) satisfying relationships, (11) financial stability, and (12) material stability. A mixed graphical network analysis was employed to analyze the related determinants, divided into four groups: (a) sociodemographic factors, (b) physical functions and health status, (c) social and family engagement, and (d) community environmental characteristics as nodes.

**Results:**

We analyzed 31 variables and identified 133 nonzero edges out of 465 potential connections in the comprehensive network. Results showed that happiness and promoting good were the two most critical indicators influencing retirees’ overall flourishing. Different flourishing indicators were also associated with various influential factors. For instance, personal characteristics, especially gender and education, emerged as central factors. Family caregiving negatively affected happiness and financial stability, whereas social engagement was positively associated with life satisfaction and meaning in life. Employment status had mixed effects, negatively associated with life satisfaction but positively associated with mental health. Community environments, such as a sense of community and neighborhood safety, generally enhanced flourishing. However, the accessibility of neighborhood resources was paradoxically associated with material stability, pointing to the complexity of environmental factors in human flourishing.

**Conclusion:**

This study provides a comprehensive network analysis that reveals intricate connections between personal, behavioral, and environmental factors, offering profound insights for targeted interventions to foster human flourishing.

**Supplementary Information:**

The online version contains supplementary material available at 10.1186/s12889-024-19466-x.

## Introduction

The concept of human flourishing has recently gained significant attention from individuals, academics, and policymakers, especially after the COVID-19 pandemic highlighted the need to support the capabilities and conditions necessary for recovering from recessions [1]. Although consensus is still being sought, flourishing is widely accepted as “the relative attainment of a state in which all aspects of a person’s life are good, including the contexts in which that person lives” [2]. This broad definition stresses that flourishing encompasses many aspects of living conditions and recognizes the importance of the external environment for individuals to pursue flourishing [2, 3]. Exploring factors associated with flourishing is especially crucial for retirees, who possess ample free time to engage in personal interests following their departure from the workforce. Comprehending how their health status, behaviors, and the surrounding environment influence their flourishing holds significant implications for policy formulation, practical applications, and scholarly research.

### Distinctions from well-being, quality of life, and holistic health

Human flourishing encompasses at least three concepts: well-being, quality of life, and overall health, but it is not limited to these terms. Well-being centers on subjective assessments of life circumstances, often measured as life satisfaction. Quality of life combines subjective feelings with objective measures that assess personal happiness and safety. Holistic health addresses physical, mental, and social health, focusing on maintaining disease-free conditions. However, human flourishing extends beyond the above concepts, integrating elements, such as life satisfaction, broad assessments for quality of life, and health-related situations, while emphasizing the crucial interactions between individuals and their environments [2, 3].

### Development of flourishing research

As flourishing is a relatively new concept, past research has focused on exploring the essential elements of human flourishing and proposing a set of measurable factors through quantitative indexes [1, 4]. For example, Seligman’s PERMA model identifies positive emotion, engagement, relationships, meaning, and achievement as the core elements people pursue for themselves, collectively defining flourishing [5]. Diener et al. (2010) developed the scale by incorporating aspects, such as life purpose, positive relationships, engagement, competence, self-esteem, and optimism, so they used a composite score to assess overall flourishing [6]. Huppert and So’s (2013) study is similar to Diener et al.’s, but it adds the elements of emotional stability and vitality [7]. Keyes’s model broadly integrates aspects of flourishing, including autonomy, environmental mastery, personal growth, social contribution, social coherence, and social integration [8, 9].

In 2017, Harvard University developed a Secure Flourish Index that outlined six critical domains in human life for flourishing, referred to in this study as VanderWeele’s domain approach. These critical domains are (1) happiness and life satisfaction, (2) physical and mental health, (3) meaning and purpose, (4) character and virtue, (5) close social relationships, and (6) material and financial stability. In this framework, each domain consists of two indicators, for a total of 12 indicators, which will be applied in this study. This shows that flourishing is not only an ideal state but a practical and inclusive concept encompassing many human experiences and aspirations [3].

After reviewing related practical articles, however, most studies on flourishing have typically focused on an overall score or a limited number of indicators. For example, Schotanus-Dijkstra et al. (2016) concentrated on flourishing only at individual levels, examining factors, such as sociodemographic characteristics; personality traits; and situational elements, such as social support and physical health [10]. Lee et al. (2022) investigated factors influencing multiple flourishing indicators among US employees, focusing solely on race/ethnicity, gender, and age [11]. The above studies have not extensively explored how different flourishing indicators interact or examined the role of broader influences between community context and individual behavior. These limitations leave a gap in understanding the dynamic interplay of multiple factors among individuals in the real world that shape our flourishing lives.

### Theoretical basis for the study

This study focused on the retired population, which completely differs from the general population. Retirement is a critical life stage characterized by significant changes in identity, social roles, and activities, with profound consequences for flourishing. Thus, the socioecological model provides a suitable framework for understanding the role of individual characteristics and environmental interactions. The model encompasses multiple levels, including (1) intrapersonal factors, such as personal traits and health behaviors; (2) interpersonal relationships with family and friends; (3) support from workplaces and organizations; (4) the community environment focusing on safety and participation; and (5) policy-level factors affecting access to essential resources [12].

Given that retirees have transitioned out of the workplace, their lives have shifted toward family and social aspects. This study focused on three levels of the socioecological model, which was divided into four types of variables, namely, (a) sociodemographic factors, (b) physical functions and health status, (c) social and family engagement, and (d) community environmental characteristics.

### Rationale for factor selection

On the basis of VanderWeele’s (2017) review of factors affecting human flourishing [3], we selected intrapersonal variables, such as gender, age, education, marital status, religious affiliation, cohabitation with children, and income. Moreover, from the perspective of maintaining functional capacity, our focus extends beyond disease and unhealthy behaviors to include sensory assessments of the pain experience, dental health, and visual and auditory functions. These health characteristics help us understand how bodily functions relate to indicators of flourishing.

For interpersonal variables, we chose social participation and family engagement factors. Retirees engaging in activities, such as social participation, volunteering, continued employment, or caregiving is common. These activities have been indicated to enhance mental health by maintaining retirees’ vitality [13, 14]. However, the effects of these activities may vary significantly across cultural contexts. For instance, in many Asian countries, caregiving is often seen as a retirees’ responsibility, a cultural expectation considerably more potent than in Western countries, which can significantly affect the health of retirees [15].

In addition, working conditions significantly influence outcomes in postretirement employment. In Germany and Switzerland, retirees who continued working after retirement reported a high life satisfaction, whereas a Korean study showed the reverse results [16]. These variations underscore the cultural differences in perceptions of postretirement activities and their effects on retirees’ flourishing.

Regarding environmental characteristics, the neighborhood environment profoundly shapes individuals’ flourishing through physical conditions and community cohesion. For example, previous studies have indicated that a city’s financial wellness and safety levels affect residents’ life satisfaction [17, 18]. A safe walking environment facilitates leisure walking, fostering interactions among residents and enhancing a sense of belonging and social cohesion, all of which contribute to great flourishing [19, 20].

### Why use Taiwanese retirees as an example

As a developed Asian region, Taiwan faces the severe challenge of a rapidly aging population. In this regard, we must actively develop policies related to healthy aging for the retired population. However, most past studies examining the relationships between environmental characteristics and human flourishing have focused on Western contexts [21]. Taiwan’s unique environment includes Chinese culture, high population density, convenient transportation, easy access to public services, and overall community safety. These environmental conditions on retired individuals may differ significantly from those in Western settings. Thus, the variations between country demographics, individual factors, and ecological characteristics underscore the need to investigate how these factors relate to retiree flourishing in Asia.

### Network analysis approach

Network analysis is a powerful tool for examining complex relationships of many variables (nodes) and their interconnections (edges). It stands out in health psychology, which supports exploring relationships between disease and behavioral patterns. For example, the Foresight Obesity Map for the UK uses network analysis to show how over 100 variables and over 300 relationships from different dimensions (e.g., biological, psychological, psychosocial, and environmental factors) converge to influence obesity [22]. This approach enables researchers to simultaneously consider and analyze multiple interacting factors, revealing the dynamic relationship between health behaviors and psychological states.

In past studies of human flourishing, such as those using VanderWeele’s domain approach, factor analysis has commonly been conducted to reduce the number of indicators and enhance model interpretability. However, this approach may ignore subtle differences among variables, resulting in the loss of critical information. By contrast, network analysis captures the nuances and the specific connections among variables, offering a detailed description of direct and indirect relationships. This approach reveals complex interactions and pathways of influence. The ability of network analysis to integrate and visualize complex data through structures, connections, and graphical representations enables a deep and adequate understanding of complex health issues.

### Feature of the study

This study aims to explore the determinants of multiple facets of flourishing indicators, particularly in the context of Asian countries, using the case of retirees. A distinctive feature of this study is its application of network analysis, which advances beyond the traditional method of aggregating indicators through factor analysis, as seen in previous studies. This study primarily aims to (1) understand the interplay and associations among flourishing indicators and (2) investigate the determinants of each flourishing indicator, including personal demographics, health issues, community characteristics, and family and social engagement.

## Methods

### Data source and participants

The Health and Living Environment Survey (HLES) was conducted by the Healthy Cities Research Center at National Cheng Kung University from April to July 2023, targeting retirees aged 50–74 who received civil service or labor insurance pensions in Taiwan. This survey used a two-stage systematic sampling method within Taiwan’s administrative framework, comprising 20 cities/counties and 358 districts/townships (excluding two outside islands). We selected districts/townships across Taiwan by levels of urbanization through stratified random sampling based on Liu’s (2006) urbanization classification [23]. Subsequent sampling of neighborhoods and participants within these areas and households was random. Finally, this survey included 17 cities/counties and 49 districts/townships, representing a significant portion of the national administrative divisions. Data were collected through face-to-face interviews with 35 trained interviewers using standardized questionnaires and protocols to ensure consistency and reliability across the data.

Ultimately, the number of valid respondents reached 1,115, surpassing the minimum sample size requirement of 1,068 participants. This requirement was calculated using Dillman’s (2000) formula, which accounts for a population heterogeneity of 0.5 and a sampling error of ± 3% at a 95% confidence interval (CI) [24]. After including all valid respondents, we excluded two individuals diagnosed with dementia, one without a flourishing measurement, and one missing a community factor. The final sample in the analysis comprised 1,111 retirees.

### Study framework

This study employed an integrated framework based on a socioecological model to probe into the complex construct of human flourishing and its determinants. Within this framework, we assessed a spectrum of factors, including four groups of 20 determinants: personal characteristics, health conditions, social and familial engagement, and community characteristics. Notably, this study did not condense flourishing by scoring them as domains or overall status but examined the individual influence of 12 indicators.

Given the high correlation and overlap of meaning among indicators, flourishing “9. Content relationships” and “10. Satisfying relationships” are combined on average as a new variable called relationships in the analysis. Consequently, the study concentrated on 11 flourishing indicators.

**Fig. 1:**
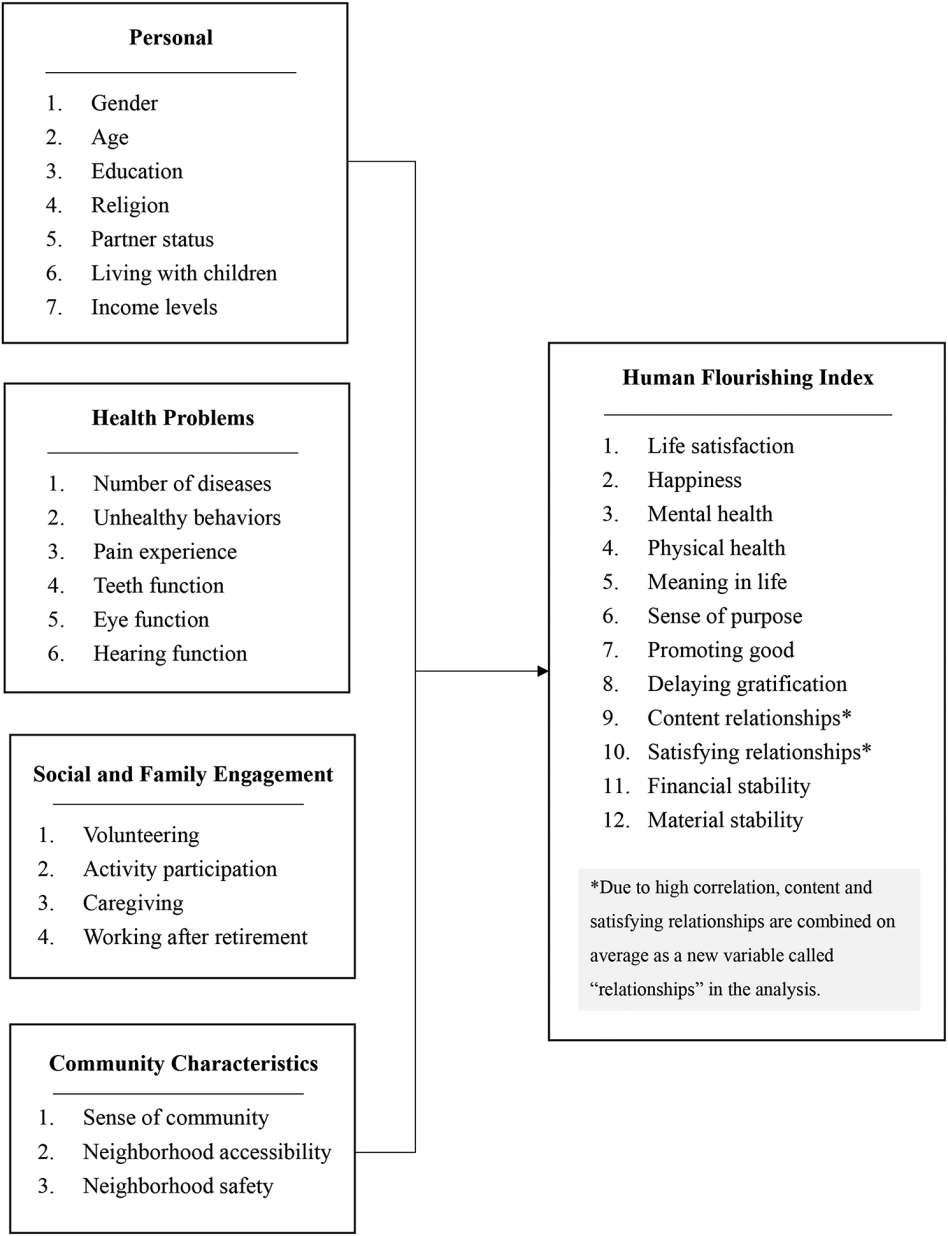
Research framework of this study

### Measurement

#### Indicators of human flourishing

We utilized the Secure Flourish Index developed by Harvard University’s Human Flourishing Program [3], which consists of 12 indicators (i.e., life satisfaction, happiness, mental health, physical health, meaning in life, sense of purpose, promoting good, delaying gratification, content relationships, satisfying relationships, financial stability, and material stability). Each item was measured on an 11-point scale, ranging from 0 to 10, as detailed in Table 1. The estimated internal consistency of the Secure Flourish Index in the study was Cronbach’s alpha (α) = 0.91.

**Table 1:** Indicators of human flourishing used in this study

Indicators	Questions	Anchors
1. Life satisfaction*	Please indicate where on the ladder you feel you personally stand right now	0 = Worse possible life, 10 = Best possible life
2. Happiness	In general, how happy or unhappy do you usually feel?	0 = Extremely Unhappy, 10 = Extremely Happy
3. Mental health	In general, how would you rate your physical health?	0 = Poor, 10 = Excellent
4. Physical health	How would you rate your overall mental health?	0 = Poor, 10 = Excellent
5. Meaning in life	Overall, to what extent do you feel the things you do are worthwhile?	0 = Not at All Worthwhile, 10 = Completely Worthwhile
6. Sense of purpose	I understand my purpose in life	0 = Strongly Disagree, 10 = Strongly Agree
7. Promoting good	I always act to promote good in all circumstances, even in challenging situations	0 = Not True of Me, 10 = Completely True of Me
8. Delaying gratification	I am always able to give up some happiness now for greater happiness later	0 = Not True of Me, 10 = Completely True of Me
9. Content relationships	I am content with my friendships and relationships	0 = Strongly Disagree, 10 = Strongly Agree
10. Satisfying relationships	My relationships are as satisfying as I would want them to be	0 = Strongly Disagree, 10 = Strongly Agree
11. Financial stability	How often do you worry about being able to meet normal monthly living expenses?	0 = Worry All the Time, 10 = Do Not Ever Worry
12. Material stability	How often do you worry about safety, food, or housing?	0 = Worry All the Time, 10 = Do Not Ever Worry, 10

*In this study, we modified VanderWeele’s (2017) question, which initially asked: “Overall, how satisfied are you with your life today?” Instead, we used the Cantril Ladder method to measure life satisfaction

#### Socioenvironmental factors

We categorized the factors influencing human flourishing into four groups: (1) personal characteristics, (2) health problems, (3) family and social engagement, and (4) community characteristics (detailed description of each variable in Supplementary Table S1).

*Personal Characteristics*

1. Gender: A binary variable as male (0) and female (1).
2. Age: A continuous variable calculated from birth year to the study period.
3. Education: Categorized into two groups, those below high school level (0) and those with high school education and above (1).
4. Religion: A binary variable for those with at least one religious belief (1) and those without any religious beliefs (0).
5. Partner status: A binary variable for those with a partner (1) and those without (0).
6. Living with children: A binary variable for those living with their son or daughter (1) or those without living with children (0).
7. Income levels: Treated as a continuous variable. The participants were asked to provide their monthly income (NT$) after retirement and were divided into eight levels, ranging from “none” to “NT$100,000 or more.”

*Health Problems*

1. Number of diseases: The respondents self-reported whether they had hypertension, heart disease, cancer, diabetes, or stroke. The study summed up these diseases as a continuous variable. Higher scores indicated more diseases.
2. Unhealthy behaviors: The respondents self-reported their habits, such as smoking, alcohol use, betel chewing, and irregular exercise. We summed up these unhealthy behaviors as a continuous variable. Higher scores indicated more unhealthy behaviors.
3. Pain experience: The respondents self-reported how often they felt severe pain in the past month. The response was rated as a continuous variable on a four-point Likert scale, ranging from 1 (never) to 4 (always).
4. Teeth function: The respondents self-reported how seriously their teeth affect their daily eating or chewing. The response was rated as a continuous variable on a four-point Likert scale, ranging from 1 (not at all) to 4 (severe).
5. Eye function: The respondents self-reported how clear their vision was. The response was rated as a continuous variable on a four-point Likert scale, ranging from 1 (strongly clear) to 4 (strongly unclear).
6. Hearing function: The respondents self-reported how clear their hearing was. The response was also rated as a continuous variable on a four-point Likert scale, ranging from 1 (strongly clear) to 4 (strongly unclear).

*Social and Family Engagement*

1. Volunteering: The respondents were asked whether they currently engage in any volunteer activities. The response was binary (yes/no).
2. Activity participation: The respondents were asked whether they participated in social groups or club activities. The response was binary (yes/no).
3. Caregiving: The respondents were asked whether they were assisting a family member with difficulties in activities of daily living (ADL) or instrumental ADL (IADL). The response was binary (yes/no).
4. Working after retirement: The respondents self-reported their current employment status. The response was binary (yes/no).

*Community Characteristics*

1. Sense of community: Assessed by an eight-item scale, capturing the ideas of needs fulfillment, group membership, influence, and emotional connection [25]. The scale was rated on a five-point Likert scale, with higher scores indicating stronger community cohesion. The Cronbach’s alpha of the scale in this study was 0.9.
2. Neighborhood accessibility: Measured by the Neighborhood Environment Walkability Scale-Abbreviated (NEWS-A), assessing the ease with which residents could walk to essential services, the availability of public transit, and the effect of the physical layout on pedestrian movement within the neighborhood [26]. The scale was rated on a four-point Likert scale, with higher scores indicating greater accessibility.
3. Neighborhood safety: Used the same NEWS-A scale but examined the interplay of traffic conditions, street infrastructure, and crime rates within the neighborhood [26]. The responses were measured on a four-point Likert scale, with higher values indicating higher perceived safety.

### Statistical methods

#### Network analysis

First, we utilized network analysis to examine the relationships among human flourishing indicators. Then, we further integrated demographic characteristics, health issues, family and social participation, and neighborhood environmental factors to identify key influences on flourishing. In this approach, variables were represented as nodes, and edges signified partial correlations controlled for other factors, with direction and strength depicted through the color and thickness of edges. Key terms in the network analysis, such as strength, betweenness, closeness, and expected influence, described the roles and importance of nodes within the network.

In detail, “Strength” was defined as the total weights of all edges connected to a node. “Betweenness” measured the frequency of a node appearing on the shortest paths between another node, with higher values indicating a significant mediating role within the network. “Closeness” examined how close a node is to all other nodes, calculating the average shortest path length from that node to other nodes. “Expected Influence” accounted for positive and negative relationships among nodes, assessing a node’s overall effect on the network.

#### Analytic strategy

We conducted network analyses following Burger’s guidelines [27]. The study analyses were performed using R version 4.0.3 within the RStudio environment, version 1.3.1095.

*Data handling and transformation*

The study had minimal missing data. That is, only two individuals were diagnosed with dementia, one without a flourishing measurement and one missing a community factor; thus, we excluded them from the network analysis. Before fitting the network, we transformed the continuous variables using a nonparametric normal transformation method from the “huge” package [28], as these variables generally exhibited skewed distributions (detailed in Supplementary Figure S1). Therefore, the associations involving continuous variables in the network were based on the transformed data.

*Assessment of overlaps and distinctiveness of flourishing indicators*

We conducted a correlation assessment of 12 flourishing indicators to identify potential topological overlaps where different variables might measure the same concept [29]. The results revealed a high correlation (*r* = 0.93) between “content relationships” and “satisfying relationships” (detailed in Supplementary Table S2). Given the conceptual similarity in the Asian context, **we thus merged them into a single indicator by averaging their scores**. Although mental and physical health, meaning and purpose, and financial and material conditions also showed high correlations, they were kept as separate indicators based on distinct conceptual differences. Therefore, only 11 flourishing indicators were used for the analysis. Furthermore, we examined all other variables in the correlation matrix and showed low correlations in Supplementary Table S3.

*Estimation method*

This study used a mixed graphical model for network analysis via the “mgm” package in software R, which was ideal for our continuous and binary data. The package employed L1 regularization, known as the least absolute shrinkage and selection operator (LASSO). This method effectively reduced extremely small edge strengths to zero, eliminating edges caused by random variations. Moreover, we employed 10-fold cross-validation as our method for model selection. This approach significantly improved the generalizability and predictability of our findings [30].

*Visualization techniques*

This study employed the “qgraph” package in software R for visualizing network models [31]. Node placement was determined using the Fruchterman–Reingold algorithm to position nodes with higher connections centrally within the network [32]. In the network graphs, **green edges signified positive correlations**,** whereas red edges denoted negative correlations**. For binary variables, such as gender, secondary education, religiosity, marital status, and living with children, a value of 1 (yes) indicated the presence of that characteristic. A red edge between gender and life satisfaction suggested a negative correlation between being female and higher life satisfaction. Furthermore, centrality analysis was conducted using the centrality plot function, with edge weights presented as standardized Z-scores. Higher scores in this analysis indicated a variable’s greater importance in the network.

*Reliability and stability of edge estimates*

To ensure the reliability of our network analysis, we used 200 nonparametric bootstrap samples to calculate edge inclusion probabilities, gauging the stability of pairwise connections. Higher inclusion probabilities indicated a more robust presence of an edge in the network. Beyond inclusion probabilities, we computed quantiles from these bootstraps for a detailed accuracy assessment of parameter estimates. However, the bootstrap quantiles from LASSO estimates differed from traditional confidence intervals because LASSO tends to bias estimates toward zero [33, 34].

## Results

### Characteristics of the participants

The final analytic sample consisted of 1,111 retirees. Among them, 52% were female, with an average age of 67.2 years, and 70% had an educational level more than high school graduates. In addition, 79% of the participants indicated they had a partner, and 49% lived with their adult children. Descriptive statistics for all the variables included in network analyses are presented in Supplementary Table S4.

### Results between flourishing indicators

**Fig. 2:**
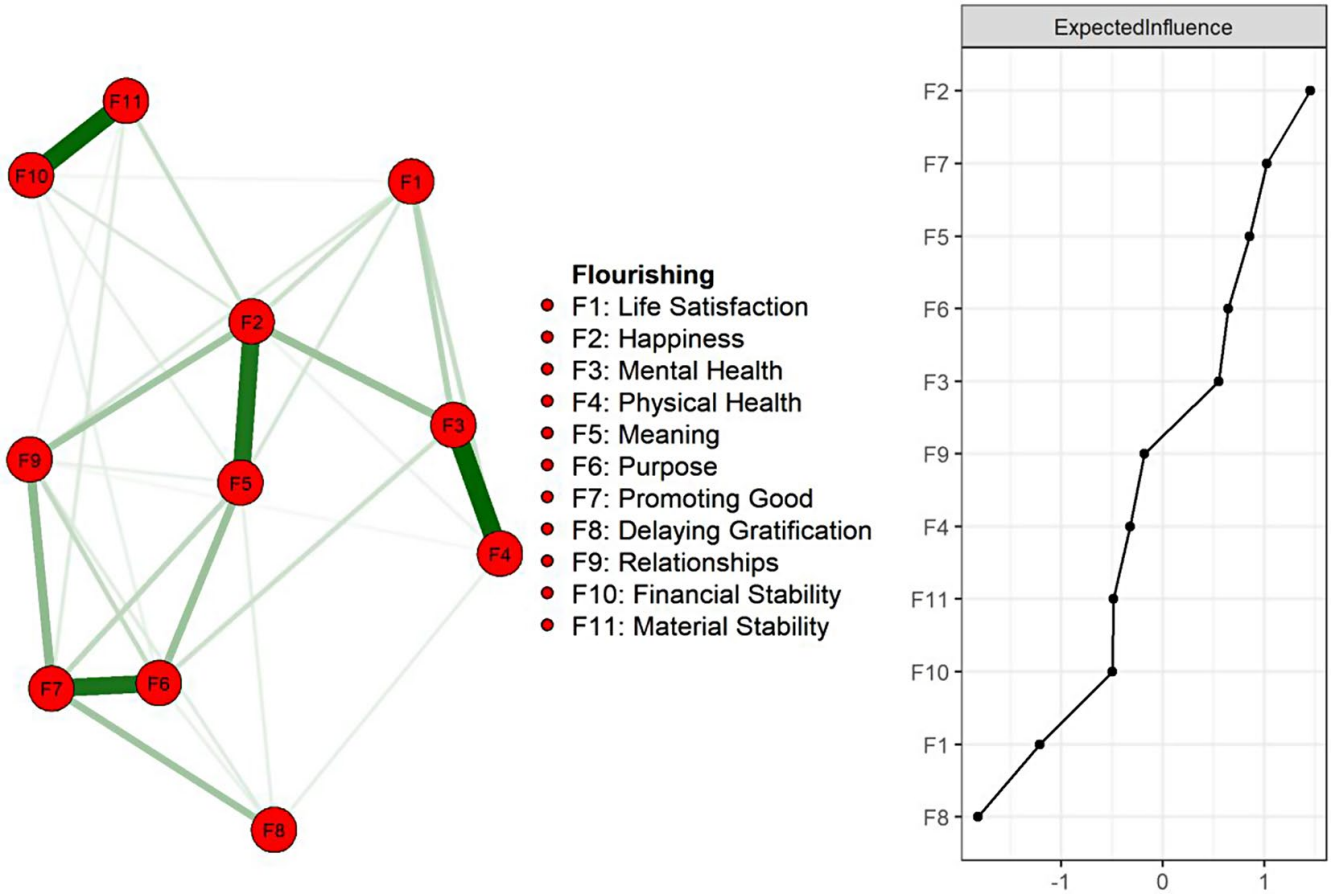
Network structure of flourishing indicators among Taiwanese retirees

Figure 2 presents the network analysis of 11 flourishing indicators. Regarding the basic characteristics of the flourishing networks, 31 of the 55 possible edges (56%) were not zero, reflecting considerable interconnectedness among indicators. According to the centrality plot (right panel of Fig. 1), F2: Happiness and F7: Prompting Good showed two of the highest expected influences. These indicators exhibited the strongest connections with other nodes within the network, implying their critical role in stimulating other connected indicators and influencing the overall flourishing status. The raw values of the centrality plot are presented in Supplementary Table S5.

#### Weighted adjacency matrices

Table 2 illustrates the weighted adjacency matrices and the predictability of each flourishing indicator. The explanatory variance of each node in the network varied significantly, ranging from approximately 27% (F8: Delaying Gratification) to 74% (F2: Happiness, F5: Meaning in Life).

#### Similarity within flourishing domains

The analysis also showed the strongest association between F3: Mental Health and F4: Physical Health (weight = 0.53) and between F10: Financial Stability and F11: Material Stability (weight = 0.52). These findings aligned with previous studies, suggesting that these variables often merge into a singular flourishing domain.

#### Heterogeneity within flourishing domains

A notable variation was observed within the flourishing domains. For example, the correlation between F2: Happiness and F5: Meaning was moderately strong (weight = 0.47), whereas F1: Life Satisfaction had a relatively weaker correlation (weight = 0.10). Similarly, F6: Sense of Purpose and F7: Promoting Good were more strongly correlated (weight = 0.48) than F5: Meaning in Life, which was relatively low (weight = 0.20). This result highlighted the interconnected yet distinct nature of the flourishing indicators.

**Table 2:** Weighted adjacency matrices and predictability of flourishing indicators*

	F1	F2	F3	F4	F5	F6	F7	F8	F9	F10	F11	*R* ^2^
F1	0.00											0.42
F2	0.10	0.00										0.74
F3	0.15	0.20	0.00									0.67
F4	0.12	0.04	0.53	0.00								0.60
F5	0.09	0.47	-	-	0.00							0.74
F6	-	-	0.10	-	0.20	0.00						0.71
F7	-	-	-	-	0.13	0.48	0.00					0.71
F8	-	-	-	0.06	0.06	0.05	0.19	0.00				0.27
F9	0.08	0.19	-	0.03	0.05	0.12	0.23	0.07	0.00			0.59
F10	0.04	0.08	-	-	0.06	0.05	-	-	-	0.00		0.54
F11	-	0.11	-	-	-	-	0.07	-	0.04	0.52	0.00	0.53

「-」: indicates no correlation between two indicators

*(F1) life satisfaction, (F2) happiness, (F3) mental health, (F4) physical health, (F5) meaning in life, (F6) sense of purpose, (F7) promoting good, (F8) delaying gratification, (F9) relationships, (F10) financial stability, and (F11) material stability

### Disentangling the flourishing determinants

**Fig. 3:**
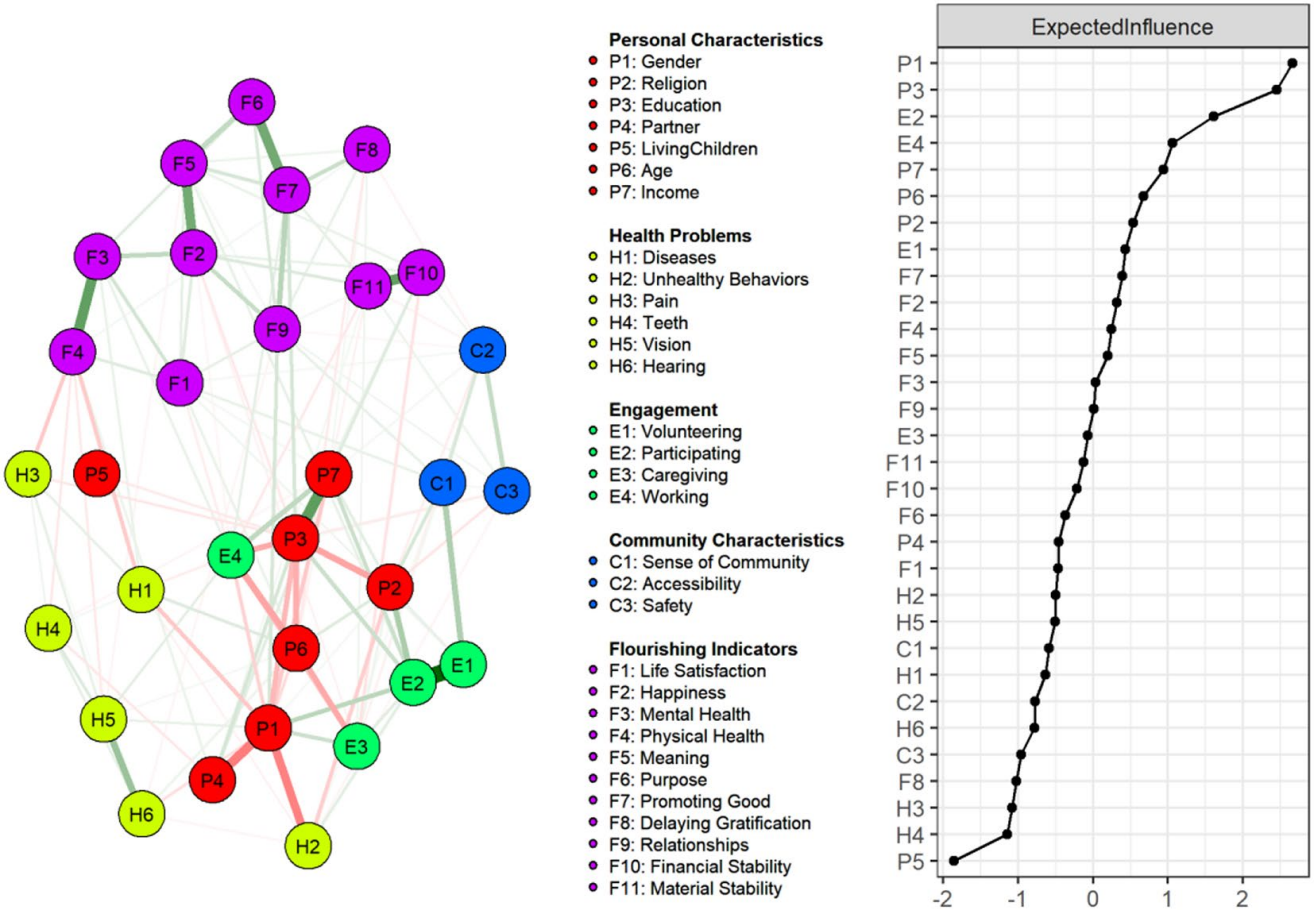
Comprehensive network structure of flourishing indicators and socioenvironmental factors

Figure 3 displays the network analysis of flourishing indicators and socioenvironmental factors, including 31 variables and 133 nonzero edges out of 465 potential connections (29% direct connectivity). The centrality plot (right panel of Fig. 2) showed the dominance of personal characteristics, such as gender and education, among the top two influential nodes. These characteristics were pivotal in connecting diverse factors, such as partner status, income, and health conditions, to flourishing. Family and social engagement also emerged as significant factors in the retirees’ perceived flourishing. Health issues and community characteristics had a relatively lesser influence. However, they exhibited strong connections with specific elements, such as the correlation between a sense of community and volunteering and the association of disease and functional ability with physical health.

**Fig. 4:**
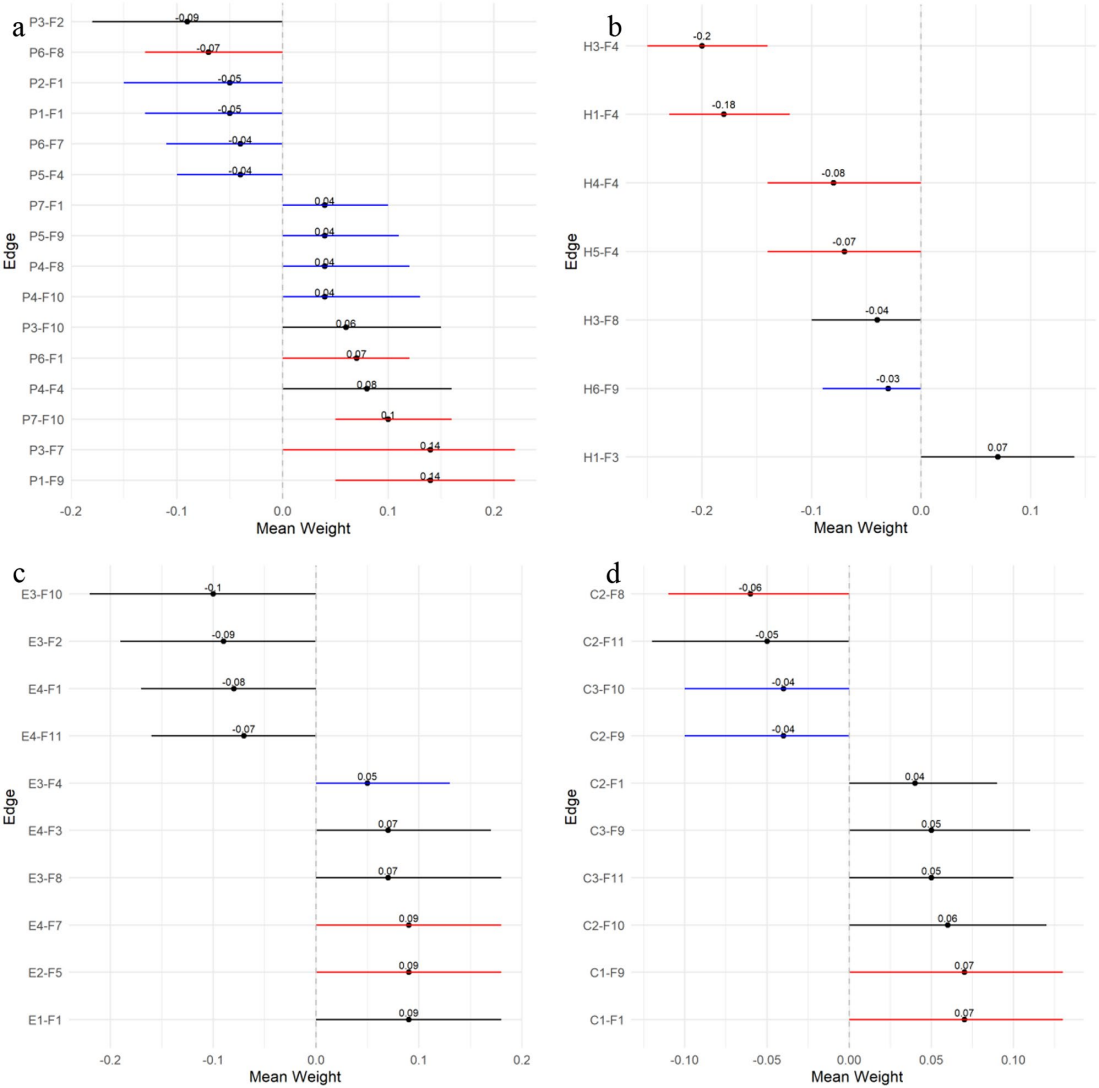
Grouped factor analysis from Table 3 on flourishing indicators. (**a**): Personal characteristics. (**b**): Health problem. (**c**): Engagement. (**d**): Community characteristics. Inclusion probabilities were color-coded: blue for 50–59%, black for 60–79%, and red for 80–100%, indicating estimated nonzero probabilities

The following sections will focus on the strongest and most reliable associations by factor groups, as illustrated in Fig. 4a–4d. Table 3 shows the results from 200 bootstrap resamples of the network data. Weighted adjacency matrices are presented in Supplementary Table S6.

#### Personal characteristics

Women reported higher satisfaction in relationships than men, the strongest and most reliable association observed (Edge (E): P1-F9, edge weight (W) = 0.14, Bootstrapped 95% quantiles (Q) = [0.05, 0.22], nonzero inclusion probability (IP) = 98%). Retirees with a high school education often showed elevated levels of virtuous characteristics (E: P3-F7, W = 0.14, Q = [0, 0.22], IP = 93%), and those with higher income correlated with increased financial security perception (E: P7-F10, W = 0.1, Q = [0.05, 0.16], IP = 96%). In addition, older retirees generally had high life satisfaction levels (E: P6-F1, W = 0.07, CI = [0, 0.12], IP = 88%), but they scored low in delaying gratification (E: P6-F8, W = − 0.07, Q = [− 0.13, 0], IP = 88%).

#### Health problems

A robust association was evident between high physical status and various health factors. Notably, less pain (E: H3-F4, W = − 0.2, Q = [− 0.26, − 0.14], IP = 100%); fewer disease (E: H1-F4, W = − 0.18, Q = [− 0.23, − 0.12], IP = 100%); and improved functional abilities, such as fewer chewing difficulties (E: H4-F4, W = − 0.08, Q = [− 0.14, 0], IP = 86%) and vision problems (E: H5-F4, W = − 0.07, Q = [− 0.14, 0], IP = 84%), were all strongly linked to improved physical health. Interestingly, a high disease presence also correlated with high self-assessed mental health (E: H1-F3, W = 0.07, Q = [0, 0.14], IP = 78%).

#### Social engagement

Within the network, most factors displayed moderate to strong stability in their nonzero associations. Current employment correlated with positive character traits (E: E4-F7, W = 0.1, Q = [0, 0.18], IP = 87%) and mental health (E: E4-F3, W = 0.08, Q = [0, 0.17], IP = 74%). However, it was negatively associated with life satisfaction (E: E4-F1, W = − 0.08, Q = [− 0.18, 0], IP = 77%) and material stability (E: E4-F11, W = − 0.07, Q = [− 0.16, 0], IP = 74%). Participation in activities positively correlated with sense of meaning (E: E2-F5, W = 0.09, Q = [0, 0.18], IP = 80%). Similarly, involvement in volunteer work was linked to high life satisfaction (E: E1-F1, W = 0.09, Q = [0, 0.18], IP = 78%). Conversely, family caregiving was associated with low financial stability perception (E: E3-F10, W = − 0.1, Q = [− 0.21, 0], IP = 78%) and less happiness (E: E3-F2, W = − 0.09, Q = [− 0.19, 0], IP = 77%).

#### Community characteristics

Analysis of community characteristics showed that great accessibility to services was linked to a low tendency for delaying gratification (E: C2-F8, W = − 0.06, Q = [− 0.11, 0], IP = 81%) and perception of material stability (E: C2-F11, W = − 0.05, Q = [− 0.12, 0], IP = 62%), but it was positively associated with financial stability (E: C2-F10, W = 0.06, Q = [0, 0.12], IP = 73%). In addition, community safety positively correlated with material stability (E: C3-F11, W = 0.05, Q = [0, 0.1], IP = 64%) and relationship satisfaction (E: C3-F9, W = 0.05, Q = [0, 0.11], IP = 72%). Sense of community showed a positive correlation with high life satisfaction (E: C1-F1, W = 0.07, Q = [0, 0.13], IP = 90%) and relationship satisfaction (E: C1-F9, W = 0.06, Q = [0, 0.13], IP = 82%).

Unhealthy behaviors showed no direct correlation with the 11-item flourishing indicators. A sense of purpose is not directly related to all other factors.

**Table 3:** Details of edge weights, inclusion probabilities, and bootstrapped 95% quantiles for pairwise associations with flourishing indicators

Indicators	Factors		Mean Weight (w)	Bootstrapped 95% Quantiles (Q)	Inclusion Probability (IP)
F1: Life Satisfaction	C1	SOC	0.07	[0, 0.13]	**0.9**
	P6	Age	0.07	[0, 0.12]	**0.88**
	E1	Volunteering	0.09	[0, 0.18]	0.78
	E4	Current Work	−0.08	[− 0.18, 0]	0.77
	C2	Accessibility	0.04	[0, 0.09]	0.61
	P7	Income	0.04	[0, 0.1]	0.56
	P2	Religion	−0.05*	[− 0.15, 0]	0.54
	P1	Gender	−0.05*	[− 0.13, 0]	0.52
F2: Happiness	P3	Education	−0.09	[− 0.18, 0]	0.78
	E3	Caregiving	−0.09	[− 0.19, 0]	0.77
F3: Mental Health	H1	Diseases	0.07	[0, 0.14]	0.78
	E4	Current Work	0.08	[0, 0.17]	0.74
F4: Physical Health	H3	Pain Experience	−0.2	[− 0.26, − 0.14]	**1**
	H1	Diseases	−0.18	[− 0.23, − 0.12]	**1**
	H4	Teeth Problem	−0.08	[− 0.14, 0]	**0.86**
	H5	Vision Problem	−0.07	[− 0.14, 0]	**0.84**
	P4	Partner	0.08	[0, 0.16]	0.74
	P5	Living with Children	−0.04*	[− 0.1, 0]	0.53
	E3	Caregiving	0.05*	[0, 0.13]	0.52
F5: Meaning in Life	E2	Activity	0.09	[0, 0.18]	**0.8**
F7: Promoting Good	P3	Education	0.14	[0, 0.22]	**0.92**
	E4	Current Work	0.1	[0, 0.18]	**0.87**
	P6	Age	−0.04*	[− 0.11, 0]	0.5
F8: Delaying Gratification	P6	Age	−0.07	[− 0.13, 0]	**0.88**
	C2	Accessibility	−0.06	[− 0.11, 0]	**0.81**
	E3	Caregiving	0.07	[0, 0.18]	0.73
	H3	Pain Experience	−0.04*	[− 0.09, 0]	0.66
	P4	Partner	0.04*	[0, 0.12]	0.51
F9: Relationships	P1	Gender	0.14	[0.05, 0.22]	**0.98**
	C1	SOC	0.06	[0, 0.13]	**0.82**
	C3	Safety	0.05	[0, 0.11]	0.72
	C2	Accessibility	−0.04	[− 0.1, 0]	0.54
	H6	Hearing Problem	−0.03	[− 0.09, 0]	0.52
	P5	Living with Children	0.04*	[0, 0.11]	0.52
F10: Financial Stability	P7	Income	0.1	[0.05, 0.16]	**0.96**
	E3	Caregiving	−0.1	[− 0.21, 0]	0.78
	C2	Accessibility	0.06	[0, 0.12]	0.73
	P3	Education	0.06	[0, 0.15]	0.62
	C3	Safety	−0.04*	[− 0.1, 0]	0.58
	P4	Partner	0.04*	[0, 0.13]	0.5
F11: Material Stability	E4	Current Work	−0.07	[− 0.16, 0]	0.74
	C2	Accessibility	−0.05	[− 0.12, 0]	0.62
	C3	Safety	0.05	[0, 0.1]	0.64

*These edges were not present in the network but were revealed in bootstrap resamples

## Discussion

This study aims to map out the intricate relationships among 11 indicators of human flourishing and related influential factors. We used network analysis to analyze how flourishing interconnects with one another and how socioenvironmental factors, including personal characteristics, health status, community features, and social behaviors, affect flourishing indicators, providing insights into the complex nature of human flourishing.

### Interrelationships between flourishing indicators

Our study identified “happiness” as a critical indicator of flourishing, aligning with Huppert’s (2013) perspective that positive emotions are essential for flourishing individuals [7]. Previous research that used domain-based approaches has revealed strong connections between the domains of “happiness” and “life satisfaction” and those of “meaning in life” and “sense of purpose.” They also found that financial and material stability tend to be negatively correlated with other areas of flourishing among Chinese and American workers [35, 36]. In this study, we further refined these relationships by confirming the associations between indicators of “happiness” and “meaning in life,” as well as indicators of “sense of purpose” and “promoting good.” Moreover, we found that financial and material stability are positively related to other measures of flourishing among Taiwanese retirees, highlighting differences in the constituents of flourishing across different groups and cultural contexts.

### Determinants of flourishing indicators

This study revealed the profound effects of personal characteristics on human flourishing. Particularly, women exhibited a stronger correlation with satisfaction in interpersonal relationships than men, likely attributed to their more extensive social networks after retirement. This condition is notably vital for meeting the relational needs of Asian women [11, 37]. Our data also indicated that age and education affect delaying gratification and life satisfaction differently. In line with socioemotional selectivity theory, younger retirees tend to focus more on future gains and delaying gratification, whereas older retirees seek immediate emotional satisfaction, often experiencing higher life satisfaction [38, 39]. Furthermore, higher educational attainment and income levels were associated with improved character development and financial stability.

Regarding health factors, our findings revealed stable and direct negative correlations between self-perceived physical health and health symptoms and weaker correlations with other flourishing indicators. These results suggest that health problems predominantly influence other flourishing indicators through perceived health, underscoring the critical role of the health dimension in determining flourishing. We also observed an intriguing phenomenon where individuals with more medical conditions tended to report better mental health than their counterparts. This could be explained by the possibility that retirees facing health challenges have developed more robust resilience and coping mechanisms, allowing them to maintain positive mental health despite physical ailments.

In addition, this study highlighted the complex interplay between social engagement, family caregiving, and employment status in influencing flourishing indicators. Social involvement and volunteering were positively associated with perceptions of a meaningful life and satisfaction. By contrast, family caregivers often reported less happiness and more financial worries, likely due to the demanding nature of caregiving [40, 41]. Interestingly, currently employed retirees tended to report low life satisfaction, consistent with the disutility of work theory, which posits that negative aspects of work contribute to this dissatisfaction [42, 43]. Moreover, our results support the notion that healthy individuals facing material insecurity might return to the workforce [44, 45], underscoring the complexity of human decision-making processes.

Our study also sheds light on the relationship between community bonds and flourishing indicators in retirees. A strong sense of community was associated with high life satisfaction. Although easy access to resources generally correlates with high life satisfaction and financial stability, we found an unexpectedly negative correlation between delaying gratification and material stability. The literature suggests that neighborhoods with good walkability and convenient transportation enhance community interaction, thereby improving life satisfaction [18, 46, 47]. Moreover, income significantly influences residential choices [48], but the ease of accessing resources might reduce the need for delaying gratification. Paradoxically, residents in resource-rich areas might still experience material insecurities, such as increased food insecurity or high housing costs, especially in high-income neighborhoods [49]. This finding highlights the complex interplay between socioeconomic factors and community characteristics.

### Applications and implications

The network analysis results from this study have direct real-world applications, particularly in designing effective and tailored interventions. For instance, the results underscore a relatively robust positive association between being female and relationship satisfaction. Therefore, if the goal is to enhance women’s overall flourishing, then improving their relationship satisfaction should be prioritized.

Moreover, this study provides a framework to understand the complex interplay of factors that influence indicators of retiree flourishing. We found that social participation significantly enhances the flourishing status. This type of social engagement directly increases the meaning of life and indirectly promotes retirees’ flourishing through spillover effects, such as participation in volunteer activities and strengthened social connections. Notably, postretirement employment also significantly influences overall flourishing status. The network analysis revealed that although postretirement employment might positively affect perceived mental health, it can simultaneously detract from life satisfaction. This dual effect illustrates that personal flourishing is influenced by the interplay of multiple indicators, some of which significantly affect the overall outcome. This insight is crucial for policy development, highlighting the need to consider a balance of factors when initiating policies to maximize the positive outcomes on retirees’ flourishing.

Finally, our research emphasizes the crucial role of community characteristics in influencing individual flourishing. The findings indicated that community safety and resource accessibility are positively associated with community cohesion, and such cohesion also enhances social participation and life satisfaction. This notion underscores the importance of improving physical aspects of the urban environment, such as walkability and safety, as critical strategies to boost residents’ flourishing. These improvements directly and indirectly enhance residents’ flourishing with their interpersonal relationships and overall life satisfaction.

### Strengths and limitations

To the best of our knowledge, this study is the first to employ network analysis to explore multilevel factors across multifaceted indicators of human flourishing, particularly among Asian retirees. We collected and constructed a representative sample of Taiwanese retirees, significantly enhancing the relevance and applicability of our findings to this demographic. Furthermore, we applied a socioecological model and carefully selected factors relevant to the Asian cultural context and retirement life to identify critical determinants and their roles in shaping flourishing. In addition, advanced network analysis techniques represent a major methodological strength of this study, allowing us to delineate detailed relationships and influence patterns among multiple variables within the flourishing network. This approach deepens our understanding of the structural dynamics of flourishing and tailors our insights to the specific needs and circumstances of retired populations.

Although this study makes a notable contribution to the application of network analysis in human flourishing research, it also has inherent limitations. First, using cross-sectional data precludes the inference of causal relationships between determinants and indicators. Future research is warranted to employ longitudinal designs to understand the causal and temporal dynamics between factors and human flourishing. Second, although we considered topological overlap in flourishing indicators, we could not disregard the potential overlap effects among other flourishing indicators that exhibit high correlations despite their conceptual differences. Third, this study did not delve into potential mediating paths or investigate moderating variables, such as gender or age-related differences, leaving a gap for further research. Fourth, using VanderWeele’s components of human flourishing may not capture the full complexity of the concept. Finally, due to limitations in the questionnaire, we did not collect data on factors such as personality traits. This unmeasured variable also deserves consideration in future research to achieve a comprehensive view of human flourishing.

### Contributions

This study makes significant contributions to methodological and academic discourse. Our findings provide an empirical basis for the ongoing scholarly debate on how to conceptualize and operationalize the concept of flourishing effectively. The results help clarify the relative importance of various indicators, enhancing our understanding of what drives human flourishing among retirees. This study also highlights the limitations of using aggregate scores to measure flourishing. It illustrates how different individual characteristics and community environments can lead to positive or negative changes in different flourishing indicators. These insights provide new perspectives for future research, particularly in exploring how multidimensional experiences in real-world society may affect flourishing and mental health. This opens up new ways of leveraging and interpreting health outcomes, potentially leading to nuanced and compelling health and social policy interventions.

## Conclusions

This study has revealed diverse factors affecting retirees’ flourishing, including personal characteristics, health status, social engagement, and community environments. The network analysis emphasized that happiness and promoting good are essential indicators for retirees’ flourishing. We also observed significant roles of gender and educational attainment and positive correlations between social participation and community cohesion in enhancing human flourishing. However, the analysis also revealed complex relationships, such as postretirement work positively affecting mental health but adversely influencing life satisfaction. These findings highlight the necessity for comprehensive and balanced considerations when developing policies and interventions for flourishing. Understanding these dynamics is essential for devising effective strategies to support retirees’ flourishing comprehensively.

### Electronic supplementary material

Below is the link to the electronic supplementary material.

Supplementary Material 1

## Data Availability

The datasets used and/or analyzed during the current study are not publicly accessible due to privacy and confidentiality constraints imposed by the data-collecting organization. However, they are available from the corresponding author upon reasonable request.
