# Supplementary material for: Exploring determinants of flourishing: a comprehensive network analysis of retirees in Taiwan
Source: BMC Public Health. 2024 Jul 19;24:1939. doi: 10.1186/s12889-024-19466-x (PMC11264847; doi:10.1186/s12889-024-19466-x)
Supplement: Supplementary file 1 — Supplementary Material 1 [file 12889_2024_19466_MOESM1_ESM.docx]

Table S1: Details of variables included in network models

| **Category** | **Variable** | **Questions** | **Scoring** | **Variable type (used in analysis)** |
| --- | --- | --- | --- | --- |
| Personal  Characteristics | 1. Gender | What is your gender? | 0=Male  1=Female | Binary |
|  | 1. Age | What is your year of birth? | Age in years | Continuous |
|  | 1. Education | What is your highest educational attainment? | 0=Below high school  1= high school and above | Binary |
|  | 1. Religion | What is your main religious belief? | 0=No religious belief  1=Yes | Binary |
|  | 1. Partner status | Do you have a partner? | 0=No partner  1=Yes | Binary |
|  | 1. Living with children | Do you live with a son or daughter? | 0=Not living with children  1=Living with children | Binary |
|  | 1. Income levels | What is your approximate monthly income after retirement? (NT$) | 1=None  2= ≤$4999  3=$5000-$14999  4=$15000-$24999  5=$25000-$39999  6=$40000-$69999  7=$70000-$99999  8=≥$100000 | Continuous |
| Health  problems | 1. Number of diseases | Have you been diagnosed by a physician with any of the following diseases? Hypertension, diabetes, heart disease, stroke or mini-stroke, cancer or malignant tumor. | 0~5 | Continuous |
|  | 1. Unhealthy behaviors | Do you have any of the following behaviors? Smoking, alcohol use, betel chewing, and irregular exercise. | 0~4 | Continuous |
|  | 1. Pain Experience | How often did they feel severe pain in past month? | 1=Never  2=Sometimes  3=Usually/Always | Continuous |
|  | 1. Teeth Function | How serious did your teeth affect your daily eating or chewing food? | 1= Excellent  2= Good  3= Poor  4= Very Poor | Continuous |
|  | 1. Eye function | How clear on your vision? | 1= Excellent  2= Good  3= Poor  4= Very Poor | Continuous |
|  | 1. Hearing function | How clear on your hearing? | 1= Excellent  2= Good  3= Poor  4= Very Poor | Continuous |
| Social and family engagement | 1. Volunteering | Are you currently serving as a volunteer? | 0=No  1=Yes | Binary |
|  | 1. Activity Participation | Are you currently participating in any club or organization activities? | 0=No  1=Yes | Binary |
|  | 1. Caregiving | Are you currently providing care for a family member? | 0=No  1=Yes | Binary |
|  | 1. Working after retirement | Are you currently employed? | 0=No  1=Yes | Binary |
| Community Characteristics | 1. Sense of Community | 1. I can get what I need in this neighborhood. 2. This neighborhood helps me fulfill my needs. 3. I feel like a member of this neighborhood. 4. I belong in this neighborhood. 5. I have a say about what goes on in my neighborhood. 6. People in this neighborhood are good at influencing each other. 7. I feel connected to this neighborhood. 8. I have a good bond with others in this neighborhood. | 1=Strongly Disagree  2= Disagree  3= Neutral  4= Agree  5=Strongly Agree  Sum up all items. Score range from 8-40. | Continuous |
|  | 1. Neighborhood Accessibility | 1. Stores are within easy walking distance of my home. 2. Parking is difficult in local shopping areas. 3. There are many places to go within easy walking distance of my home. 4. It is easy to walk to a transit stop (bus, train) from my home. 5. The streets in my neighborhood are hilly, making my neighborhood difficult to walk in. 6. There are major barriers to walking in my local area that make it hard to get from place to place (for example, freeways, railway lines, rivers). | 1=Strongly Disagree  2= Disagree  3= Agree  4=Strongly Agree  Sum up all items. Score range from 6-24. | Continuous |
|  | 1. Neighborhood Safety | 1. There is so much traffic along nearby streets that it makes it difficult or unpleasant to walk in my neighborhood. 2. The speed of traffic on most nearby streets is usually slow (30 mph or less). 3. Most drivers exceed the posted speed limits while driving in my neighborhood. 4. My neighborhood streets are well lit at night. 5. Walkers and bikers on the streets in my neighborhood can be easily seen by people in their homes. 6. There are crosswalks and pedestrian signals to help walkers cross busy streets in my neighborhood. 7. There is a high crime rate in my neighborhood. 8. The crime rate in my neighborhood makes it unsafe to go on walks during the day. 9. The crime rate in my neighborhood makes it unsafe to go on walks at night. | 1=Strongly Disagree  2= Disagree  3= Agree  4=Strongly Agree  Sum up all items. Score range from 8-32. | Continuous |

Table S2: Correlation matrix of 12-item Flourishing index

| **Question/statement** | **F1** | **F2** | **F3** | **F4** | **F5** | **F6** | **F7** | **F8** | **F9** | **F10** | **F11** | **F12** |
| --- | --- | --- | --- | --- | --- | --- | --- | --- | --- | --- | --- | --- |
| F1. Please indicate where on the ladder you feel you personally stand right now ? | **1** |  |  |  |  |  |  |  |  |  |  |  |
| F2. In general, how happy or unhappy do you usually feel? | **0.59** | **1** |  |  |  |  |  |  |  |  |  |  |
| F3. In general, how would you rate your physical health? | 0.56 | 0.69 | **1** |  |  |  |  |  |  |  |  |  |
| F4. How would you rate your overall mental health? | 0.52 | 0.59 | **0.75** | **1** |  |  |  |  |  |  |  |  |
| F5. Overall, to what extent do you feel the things you do in your life are worthwhile? | 0.57 | 0.82 | 0.63 | 0.55 | **1** |  |  |  |  |  |  |  |
| F6. I understand my purpose in life | 0.49 | 0.64 | 0.60 | 0.53 | **0.71** | **1** |  |  |  |  |  |  |
| F7. I always act to promote good in all circumstances, even in difficult and challenging situations | 0.45 | 0.61 | 0.54 | 0.48 | 0.67 | 0.76 | **1** |  |  |  |  |  |
| F8. I am always able to give up some happiness now for greater happiness later | 0.16 | 0.27 | 0.22 | 0.24 | 0.29 | 0.31 | **0.33** | **1** |  |  |  |  |
| F9. I am content with my friendships and relationships | 0.48 | 0.64 | 0.51 | 0.46 | 0.63 | 0.63 | 0.65 | 0.29 | **1** |  |  |  |
| F10. My relationships are as satisfying as I would want them to be | 0.48 | 0.64 | 0.52 | 0.48 | 0.65 | 0.65 | 0.65 | 0.28 | **0.93** | **1** |  |  |
| F11. How often do you worry about being able to meet normal monthly living expenses? | 0.40 | 0.55 | 0.43 | 0.39 | 0.51 | 0.44 | 0.43 | 0.21 | 0.40 | 0.43 | **1** |  |
| F12. How often do you worry about safety, food, or housing? | 0.35 | 0.50 | 0.36 | 0.35 | 0.48 | 0.40 | 0.42 | 0.18 | 0.40 | 0.42 | **0.70** | 1 |

Table S3: Correlation matrix of all variables in network analysis

| **Group** | **Personal Characteristics** | | | | | | | **Community Characteristics** | | | **Health Problems** | | | | | | **Family and Social Engagement** | | | |
| --- | --- | --- | --- | --- | --- | --- | --- | --- | --- | --- | --- | --- | --- | --- | --- | --- | --- | --- | --- | --- |
| **Variables** | Gender | Religion | Edu | Partner | Living | Age | Income | SOC | Access | Safe | Disease | Behavior | Pain | Teeth | Eye | Ear | Volunteer | Activity | Caregiving | Work |
| Gender | 1 |  |  |  |  |  |  |  |  |  |  |  |  |  |  |  |  |  |  |  |
| Religion | .08 | 1 |  |  |  |  |  |  |  |  |  |  |  |  |  |  |  |  |  |  |
| Edu | -.15 | -.09 | 1 |  |  |  |  |  |  |  |  |  |  |  |  |  |  |  |  |  |
| Partner | -.2 | 0 | .14 | 1 |  |  |  |  |  |  |  |  |  |  |  |  |  |  |  |  |
| Living | .02 | 0 | -.08 | .04 | 1 |  |  |  |  |  |  |  |  |  |  |  |  |  |  |  |
| Age | -.04 | .09 | -.18 | 0 | -.06 | 1 |  |  |  |  |  |  |  |  |  |  |  |  |  |  |
| Income | -.18 | .03 | .37 | .14 | -.06 | -.12 | 1 |  |  |  |  |  |  |  |  |  |  |  |  |  |
| SOC | .03 | .1 | .03 | .03 | .03 | .03 | .05 | 1 |  |  |  |  |  |  |  |  |  |  |  |  |
| Access | -.03 | -.05 | .04 | .03 | .03 | 0 | .05 | .17 | 1 |  |  |  |  |  |  |  |  |  |  |  |
| Safe | .04 | .03 | .02 | -.02 | -.04 | .02 | .04 | .05 | .02 | 1 |  |  |  |  |  |  |  |  |  |  |
| Disease | -.1 | .01 | -.04 | -.05 | .06 | .15 | -.06 | -.01 | 0 | .04 | 1 |  |  |  |  |  |  |  |  |  |
| Behavior | -.28 | -.1 | .04 | .04 | -.04 | -.01 | .11 | .06 | 0 | -.04 | -.05 | 1 |  |  |  |  |  |  |  |  |
| Pain | .05 | -.03 | -.14 | -.05 | .07 | .07 | -.13 | -.05 | -.01 | -.01 | .18 | -.04 | 1 |  |  |  |  |  |  |  |
| Teeth | -.05 | -.04 | -.08 | -.09 | 0 | .08 | -.11 | -.1 | -.06 | .03 | .15 | .03 | .18 | 1 |  |  |  |  |  |  |
| Eye | .03 | 0 | -.06 | -.03 | .04 | .09 | -.04 | -.04 | .02 | -.01 | .09 | -.05 | .2 | .22 | 1 |  |  |  |  |  |
| Ear | -.08 | -.02 | -.04 | -.02 | .03 | .11 | -.04 | -.1 | -.03 | .04 | .07 | -.06 | .16 | .2 | .4 | 1 |  |  |  |  |
| Volunteer | .1 | .11 | .04 | .02 | .04 | .05 | .04 | .26 | 0 | .03 | .01 | 0 | -.04 | -.09 | -.01 | -.05 | 1 |  |  |  |
| Activity | .1 | .14 | .12 | -.01 | -.01 | .03 | .15 | 0.2 | 0 | .04 | 0 | .05 | -.07 | -.09 | 0 | -.03 | .45 | 1 |  |  |
| Caregiving | .1 | .04 | .01 | .01 | .06 | -.17 | .03 | .07 | -.04 | .02 | -.02 | -.02 | -.03 | -.05 | -.04 | -.05 | .06 | .08 | 1 |  |
| Work | -.07 | -.01 | -.02 | 0 | .01 | -.2 | .14 | -.01 | .06 | .03 | -.07 | .02 | -.03 | 0 | .05 | 0 | -.05 | 0 | -.04 | 1 |

Table S3: Correlation matrix of all variables in network analysis (continued)

| **Group** | **Personal Characteristics** | | | | | | | **Community Characteristics** | | | **Health Problems** | | | | | | **Family and Social Engagement** | | | |
| --- | --- | --- | --- | --- | --- | --- | --- | --- | --- | --- | --- | --- | --- | --- | --- | --- | --- | --- | --- | --- |
| **Variables** | Gender | Religion | Edu | Partner | Living | Age | Income | SOC | Access | Safe | Disease | Behavior | Pain | Teeth | Eye | Ear | Volunteer | Activity | Caregiving | Work |
| Life Satisfaction | -.03 | -.02 | .12 | .09 | -.01 | .06 | .15 | .27 | .11 | .01 | -.13 | .05 | -.2 | -.19 | -.14 | -.11 | .16 | .11 | 0 | -.03 |
| Happiness | .01 | -.01 | .08 | .09 | 0 | .02 | .15 | .26 | .1 | .03 | -.17 | .08 | -.25 | -.24 | -.16 | -.17 | .16 | .15 | -.01 | 0 |
| Mental Health | -.01 | .01 | .13 | .06 | -.03 | -.02 | .19 | .25 | .09 | -.01 | -.14 | .08 | -.24 | -.21 | -.16 | -.19 | .12 | .13 | .03 | .07 |
| Physical Health | -.02 | 0 | .13 | .12 | -.06 | -.04 | .18 | .23 | .07 | -.02 | -.26 | .1 | -.36 | -.28 | -.24 | -.21 | .09 | .09 | .06 | .07 |
| Meaning in Life | .05 | .01 | .08 | .07 | -.01 | 0 | .13 | .27 | .09 | .04 | -.17 | .03 | -.2 | -.26 | -.17 | -.16 | .17 | .2 | .06 | -.01 |
| Sense of Purpose | .04 | .03 | .14 | .08 | .01 | -.01 | .13 | .24 | .13 | -.02 | -.13 | .03 | -.17 | -.23 | -.11 | -.15 | .14 | .18 | .09 | .03 |
| Promoting Good | .01 | .02 | .17 | .09 | 0 | -.06 | .14 | .24 | .1 | .02 | -.12 | .02 | -.17 | -.22 | -.12 | -.14 | .15 | .19 | .09 | .07 |
| Delaying Gratification | -.03 | -.04 | .11 | .08 | .01 | -.11 | .12 | .05 | -.03 | .04 | -.04 | -.01 | -.14 | -.08 | -.07 | -.05 | .07 | .04 | .09 | .03 |
| Content Relationships | .09 | .04 | .06 | .06 | .03 | .03 | .09 | .26 | .05 | .04 | -.1 | .04 | -.19 | -.19 | -.11 | -.17 | .17 | .16 | .07 | .01 |
| Satisfying Relationships | .1 | .04 | .06 | .08 | .02 | .03 | .09 | .28 | .05 | .04 | -.08 | .03 | -.21 | -.2 | -.12 | -.17 | .18 | .17 | .05 | .01 |
| Financial Stability | -.03 | -.01 | .16 | .14 | -.02 | .04 | .19 | .19 | .12 | -.02 | -.09 | .04 | -.2 | -.11 | -.09 | -.12 | .08 | .12 | -.08 | -.02 |
| Material Stability | -.03 | .01 | .1 | .12 | -.02 | .04 | .1 | .13 | .02 | -.04 | -.08 | .05 | -.15 | -.15 | -.11 | -.12 | .06 | .09 | -.05 | -.05 |

Figure S1: Distribution of continuous variables in network analysis


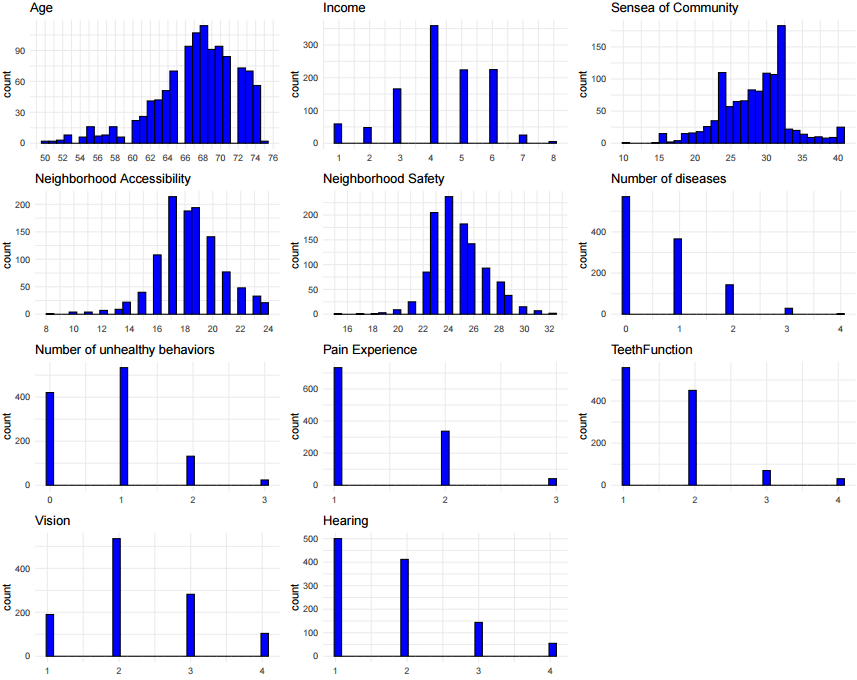

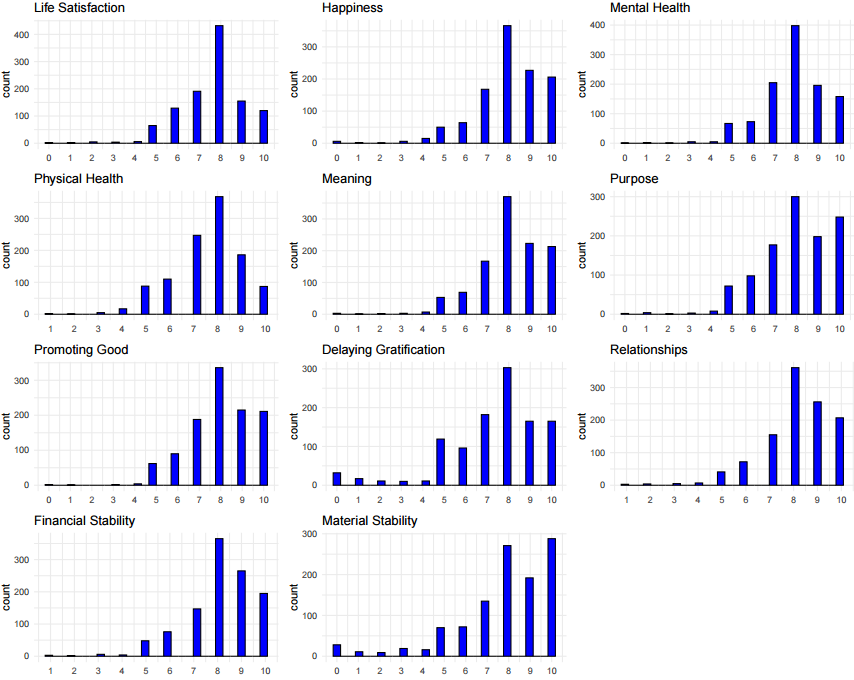


Table S4: Descriptive statistics for all variables in network analysis

|  | **level** | **label** | **Overall** | |
| --- | --- | --- | --- | --- |
| N=1111 |  |  | n | % |
| Gender | 0 | Male | 533 | 48.0 |
|  | 1 | Female | 578 | 52.0 |
| Religion | 0 | No | 190 | 17.1 |
|  | 1 | Yes | 921 | 82.9 |
| Education | 0 | below high school | 333 | 30.0 |
|  | 1 | high school and above | 778 | 70.0 |
| Partner Status | 0 | No | 236 | 21.2 |
|  | 1 | Yes | 875 | 78.8 |
| Living with Children | 0 | No | 567 | 51.0 |
|  | 1 | Yes | 544 | 49.0 |
| Income level | 1 | None | 59 | 5.3 |
|  | 2 | ≤4999 | 48 | 4.3 |
|  | 3 | 5000-14999 | 166 | 14.9 |
|  | 4 | 15000-24999 | 359 | 32.3 |
|  | 5 | 25000-39999 | 224 | 20.2 |
|  | 6 | 40000-69999 | 225 | 20.3 |
|  | 7 | 70000-99999 | 25 | 2.3 |
|  | 8 | ≥100000 | 5 | 0.5 |
| Number of diseases | 0 | None | 571 | 51.4 |
|  | 1 | One | 366 | 32.9 |
|  | 2 | Two | 143 | 12.9 |
|  | 3 | Three | 29 | 2.6 |
|  | 4 | Four | 2 | 0.2 |
| Number of unhealthy behaviors | 0 | None | 421 | 37.9 |
|  | 1 | One | 534 | 48.1 |
|  | 2 | Two | 132 | 11.9 |
|  | 3 | Three | 24 | 2.2 |
| Pain Experience | 1 | Never | 733 | 66.0 |
|  | 2 | Sometimes | 337 | 30.3 |
|  | 3 | Usually/Always | 41 | 3.7 |
| Teeth Function | 1 | Excellent | 559 | 50.3 |
|  | 2 | Good | 451 | 40.6 |
|  | 3 | Poor | 70 | 6.3 |
|  | 4 | Very Poor | 31 | 2.8 |
| Vision | 1 | Excellent | 190 | 17.1 |
|  | 2 | Good | 535 | 48.2 |
|  | 3 | Poor | 282 | 25.4 |
|  | 4 | Very Poor | 104 | 9.4 |
| Hearing | 1 | Excellent | 500 | 45.0 |
|  | 2 | Good | 412 | 37.1 |
|  | 3 | Poor | 144 | 13.0 |
|  | 4 | Very Poor | 55 | 5.0 |
| Volunteering | 0 | No | 721 | 64.9 |
|  | 1 | Yes | 390 | 35.1 |
| Activity Participation | 0 | No | 646 | 58.1 |
|  | 1 | Yes | 465 | 41.9 |
| Family Caregiving | 0 | No | 932 | 83.9 |
|  | 1 | Yes | 179 | 16.1 |
| Current Employment | 0 | No | 827 | 74.4 |
|  | 1 | Yes | 284 | 25.6 |

|  | Mean | SD | Min | Max |
| --- | --- | --- | --- | --- |
| Age | 67.18 | 4.69 | 50 | 75 |
| Sense of Community | 28.47 | 4.64 | 10 | 40 |
| Neighborhood Accessibility | 18.36 | 2.33 | 8 | 24 |
| Neighborhood Safety | 24.74 | 2.15 | 15 | 32 |
| Life Satisfaction | 7.68 | 1.48 | 0 | 10 |
| Happiness | 8.03 | 1.60 | 0 | 10 |
| Mental Health | 7.90 | 1.45 | 0 | 10 |
| Physical Health | 7.56 | 1.45 | 1 | 10 |
| Meaning in Life | 8.09 | 1.50 | 0 | 10 |
| Sense of Purpose | 8.01 | 1.64 | 0 | 10 |
| Promoting Good | 8.03 | 1.49 | 0 | 10 |
| Delaying Gratification | 7.31 | 2.25 | 0 | 10 |
| Content Relationships | 8.14 | 1.47 | 1 | 10 |
| Satisfying Relationships | 8.12 | 1.45 | 1 | 10 |
| Financial Stability | 7.79 | 2.27 | 0 | 10 |
| Material Stability | 7.82 | 2.23 | 0 | 10 |

Table S5: The raw values of centrality plot on flourishing indicators

**Strength** is defined as total weights of all edges connected to a node. **Betweenness** measures the frequency of a node appears on the shortest paths between another node, with higher values indicating a significant mediating role within the network. **Closeness** evaluates how close a node is to all other nodes, calculating the average shortest path length from that node to other nodes. **Expected Influence** accounts for both positive and negative relationships between nodes, assessing a node's overall effect in the network. In our analysis, as all edges are positive, Strength and Expected Influence are equivalent. All these centrality metrics incorporate edge weights.

| **Node** | **NodeName** | **Betweenness** | **Closeness** | **Strength** | **Expected Influence** |
| --- | --- | --- | --- | --- | --- |
| F1 | Life Satisfaction | 0 | 0.007 | 0.573 | 0.573 |
| F2 | Happiness | 17 | 0.013 | 1.200 | 1.200 |
| F3 | Mental Health | 8 | 0.010 | 0.988 | 0.988 |
| F4 | Physical Health | 0 | 0.009 | 0.782 | 0.782 |
| F5 | Meaning in Life | 5 | 0.012 | 1.060 | 1.060 |
| F6 | Sense of Purpose | 7 | 0.010 | 1.010 | 1.010 |
| F7 | Promoting Good | 9 | 0.010 | 1.100 | 1.100 |
| F8 | Delaying Gratification | 0 | 0.007 | 0.430 | 0.430 |
| F9 | Relationships | 2 | 0.010 | 0.815 | 0.815 |
| F10 | Financial Stability | 0 | 0.007 | 0.741 | 0.741 |
| F11 | Material Stability | 9 | 0.007 | 0.744 | 0.744 |

Table S6: weighted adjacency matrices of all factors associated with flourishing indicators

| **Group** | **Personal Characteristics** | | | | | | | **Community Characteristics** | | | **Health Problems** | | | | | | **Engagement** | | | |
| --- | --- | --- | --- | --- | --- | --- | --- | --- | --- | --- | --- | --- | --- | --- | --- | --- | --- | --- | --- | --- |
| **Variables** | Gender | Religion | Edu | Partner | Living | Age | Income | SOC | Access | Safe | Disease | Behavior | Pain | Teeth | Eye | Ear | Volunteer | Activity | Caregiving | Work |
| Life Satisfaction |  |  |  |  |  | .07 | .04 |  |  |  |  |  |  |  |  |  | .05 |  |  | −.06 |
| Happiness |  |  | −.07 |  |  |  |  |  |  |  |  |  |  |  |  |  |  |  | -.05 |  |
| Mental Health |  |  |  |  |  |  |  |  |  |  | .07 |  |  |  |  |  |  |  |  | .07 |
| Physical Health |  |  |  | .06 |  |  |  |  |  |  | −.17 |  | −.18 | −.07 | −.07 |  |  |  |  |  |
| Meaning in Life |  |  |  |  |  |  |  |  |  |  |  |  |  |  | -.04* |  |  | .08 |  | −.03* |
| Sense of Purpose |  |  |  |  |  |  |  |  |  |  |  |  |  |  |  |  |  |  |  |  |
| Promoting Good |  |  | .13 |  |  |  |  |  |  |  |  |  |  |  |  |  |  |  |  | .08 |
| Delaying Gratification |  |  |  |  |  | −.07 | .04* |  |  |  |  |  |  |  |  |  |  |  | .05 |  |
| Relationships | .13 |  |  |  |  |  |  |  |  |  |  |  |  |  |  | −.04 |  |  |  |  |
| Financial Stability |  |  | .06 |  |  |  | .10 |  |  |  |  |  |  |  |  |  |  |  | −.09 |  |
| Material Stability |  |  |  | .04* |  |  | −.05* |  |  |  |  |  |  |  |  |  |  |  |  | −.07 |
| *These edges were not present in the bootstrap resamples but revealed in the our network result. | | | | | | | | | | | | | | | | | | | | |
